# The Saphenous Vein Graft: Can a Frog Become a Princess?

**DOI:** 10.3390/medicina60121915

**Published:** 2024-11-21

**Authors:** Antonio Maria Calafiore, Sotirios Prapas, Ignazio Condello, Konstantinos Katsavrias, Giuseppe Nasso, Mario Gaudino

**Affiliations:** 1 1st Department of Cardiac Surgery, Henry Dunant Hospital, Leof. Mesogeion 107, 11526 Athens, Greece; am.calafiore@gmail.com (A.M.C.); sotiriosprapas@gmail.com (S.P.); kkatsavrias@yahoo.com (K.K.); 2 Department of Cardiac Surgery, Anthea Hospital GVM Care and Research, Via Camillo Rosalba 35/37, 70124 Bari, Italy; gnasso@libero.it; 3 Department of Cardiothoracic Surgery, Weill Cornell Medicine, 1300 York Ave., New York, NY 10065, USA; mfg9004@med.cornell.edu

**Keywords:** saphenous vein graft, nitric oxide, composite graft, shear stress

## Abstract

The saphenous vein graft (SVG) has been a cornerstone of coronary bypass surgery, but its long-term patency is limited by accelerated atherosclerosis. Recent advancements, including the no-touch technique and the use of SVG as a limb of the left internal thoracic artery (LITA), have shown promise in improving outcomes. Both approaches enhance nitric oxide (NO) availability, a key factor in promoting endothelial stability and arterial-like behavior in the SVG. Among these, the LITA-SVG connection may offer superior long-term benefits due to sustained NO supplementation. This paper argues that the SVG, with proper strategies, can indeed achieve outcomes comparable to arterial grafts.

## 1. Introduction

While it is true that surgical myocardial revascularization became popular through the use of the saphenous vein graft (SVG), it is also true that the limitations of using the SVG were evident from the beginning [1]. The SVG had a high failure rate and was subject to an accelerated atherosclerotic process that reduced the patency rate 10 years after surgery to less than 70% [2,3]. In the 1980s, it was shown that the patency rate of the left internal thoracic artery (LITA) grafted into the left anterior descending artery (LAD) was not only better than that of the SVG [3], but that this anatomical aspect also resulted in a better long-term outcome [4].

In the 1990s and 2000s, there was a trend to increase the number of arterial conduits (ACs) with the idea that grafting at least the circumflex artery with an AC could provide better long-term results. However, this assumption has not been proven by randomized controlled trials [5] and the use of two or more ACs is now limited to no more than 20% of patients.

## 2. Surgical Strategies to Improve the Outcome of Saphenous Vein Graft

As the SVG remained the most commonly used coronary graft, various teams in the 2010s attempted to better analyze the SVG to improve its outcomes. The strategies went in two directions.

The first focused on a different harvesting technique. The SVG was harvested together with the surrounding fat (no-touch technique), left in situ for as long as possible and stored in heparinized blood after harvesting [6]. The advantages were less traumatization of the vein, better preservation of the endothelium and thus better preservation of endothelial nitric oxide synthase (eNOS) and higher availability of nitric oxide (NO), suggesting a better patency rate. Other theories were focused on the preservation of the vasa vasorum, which could contribute to the integrity of the vein wall [7], on the mechanical support provided by the surrounding fat [8] or on the secretion of not-well-identified adipocyte-relaxing factors [8]. Clinical results of no-touch SVG showed a high patency rate of SVG after 16 years [9] in a propensity-matched study and at 8.5 years in a small RCT [10].

The second strategy was focused on the use of the SVG as a limb of the LITA [11]. Compared to the right ITA as a Y-graft from the LITA, this technique showed similar outcomes after 10 years in terms of patency rate (SVG and right ITA), freedom from death of any cause and cardiac death in a propensity-matched [12] study and in a randomized controlled trial [13]. The reasons for these improved results were the reduction in mechanical stress, the exposure of the endothelium of the SVG to protective substances produced by the LITA, such as nitric oxide, and the reduction in complications associated with aortic side clamping, such as stroke or aortic dissection [11].

## 3. The Unifying Theory

So, we have two different techniques with which we could improve the patency rate of the SVG. We have identified the frog (the SVG with suboptimal rate after 10 years) that became a princess (the SVG with high patency rate after 10 years). But how was the prince able to make a seemingly impossible change? It is unlikely that the same goal can be achieved with two such different strategies. Therefore, we need to look for a unifying theory that can explain how the SVG can improve its long-term patency rate when the techniques are so far apart.

Let us take a step back. Like all other vessels, the SVG is lined by the vascular endothelium, the largest organ in our body with a surface area of 400 to 1000 square meters and a weight of 1.0 to 1.8 kg. The vascular endothelium is formed by endothelial cells (ECs), which are the brain of our vessels. They are subjected to endoluminal forces, but by far the most important is shear stress (SS), a frictional force between the circulating blood and the vessel wall that is directly proportional to blood viscosity and volumetric blood flow and inversely proportional to the cube of the radius, and that controls almost all functions of the ECs. The variations in SS, which can change due to different patterns of blood flow, are sensed by a complex mechanotransduction mechanism. The ECs sense the hemodynamic forces generated by the blood flow in the lumen of the vessel they cover via mechanosensors and mechanosensitive complexes and adapt to the new environment if necessary. In humans, the SS in a straight artery is between 10 and 70 dynes/cm^2^. In branches, bifurcations and the inner curves of the artery, the flow is disturbed, and the net value of SS is always below 4 dynes/cm^2^. In the venous system, SS is between 1 and 6 dynes/cm^2^.

The aim of continuous adaptation to the flow rate in the vessel is to keep the SS ≥ 10 dynes/cm^2^. This is the accepted threshold to maintain ECs in a quiescent state that induces the expression of various atheroprotective and antithrombogenic products that have antioxidant, anti-inflammatory, anticoagulant and antiapoptotic functions. In contrast, disturbed flow (non-laminar, turbulent, oscillating, etc.), such as at arterial bifurcation points and bends, i.e., in areas exposed to low shear stress (<10 dyne/cm^2^), induces the expression of a number of atherogenic and thrombogenic genes (pro-oxidative, pro-inflammatory, pro-coagulant and pro-apoptotic), increases the proliferation of VECs and modulates of the phenotype of vascular smooth muscle cells (VSMCs) towards synthesis.

**Figure 1:**
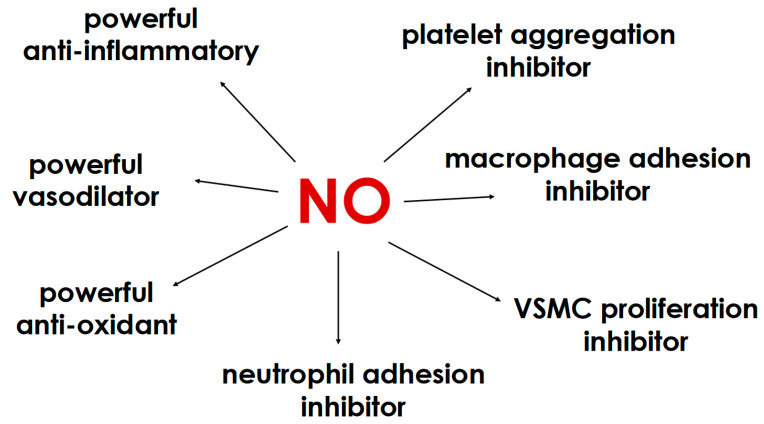
Nitric oxide protects the quiescence of vascular endothelial cells through a number of mechanisms. NO, nitric oxide; VSMC, vascular smooth muscle cell.

The ECs keep the SS stable and respond to changes in blood flow by regulating the secretion of vasodilators, such as nitric oxide (NO), and vasoconstrictors, such as endothelin-1. The vessel enlarges when blood flow increases and vice versa. In healthy blood vessels, NO contributes to the regulation of blood flow and blood pressure and inhibits the activation and aggregation of platelets as well as the adhesion and migration of leukocytes (Figure 1). Important molecular events in atherogenesis such as the oxidative modification of lipoproteins and phospholipids, the activation of ECs and the infiltration/activation of macrophages are promoted by vascular oxidative stress and inhibited by endothelial NO. SS, when lower than 10 dynes/cm^2^, downregulates the expression of endothelial nitric oxide synthase (eNOS), which generates NO and plays a critical role in maintaining coronary arterial vasodilation. Loss of eNOS reduces NO production and leads to a graded impairment of the normal dilatory response of coronary arteries to endothelium-dependent vasodilators and ultimately to vasospasm. Low SS is then a dangerous situation in the arterial system, especially for the coronary arteries, and NO availability is a crucial point that allows the arterial vessels to maintain an SS of more than 10 dynes/cm^2^ and keep the ECs quiescent.

These concepts also apply to the arterial conduits (ACs) that we use for coronary revascularization. Both the LITA and the radial artery (RA) produce NO and endothelin-1, albeit to different extents [14,15]. The LITA has a small caliber and requires relatively low internal blood flow to maintain SS ≥ 10 dynes/cm^2^. In contrast, the RA is larger than the LITA and requires a much higher blood flow to maintain SS ≥ 10 dynes/cm^2^.

But what happens when the SVG is inserted into the coronary circulation with aortic proximal anastomosis?

In its native position, the SVG has a lower production of NO than the ACs. Its caliber is large, the flow rate is not high and there is no need to change its diameter to modify the SS, which remains low at about 2 dynes/cm^2^. Assuming that the endothelium of the SVG remains intact after harvesting, the NO is not sufficient to keep the ECs of the SVG quiescent in the arterial position and its inhibitory effect on platelet aggregation is absent at the moment when it is most needed, namely in the very early phase after surgery. The SS of the SVG remains low because the flow rate, even if increased, cannot be high enough for the SS to reach the arterial level. When the SVG is then anastomosed to the aorta, it lacks sufficient NO production, has a low SS and, in other words, remains in an atherosclerotic profile. It has been reported that SS in SVG proximally anastomosed to the aorta is still 5 dynes/cm^2^ 1 year after surgery. If the SVG survives the early phase, it still remains exposed to accelerated atherosclerosis and later failure.

According to what has been written so far, a possible solution could be to supplement NO so that SVG partially or completely restores ECs’ quiescence, assuming over time an arterial phenotype.

When the SVG is harvested with a pedicle (no-touch technique), the adipose tissue around the vein acts as an endocrine organ, producing a variety of substances. NO is one of them, and can diffuse to VSMCs and thus induce vasorelaxation even in SVGs with a denudated endothelium [16]. NO can reach the ECs either by direct diffusion or via the vasa vasorum [17].

**Figure 2:**
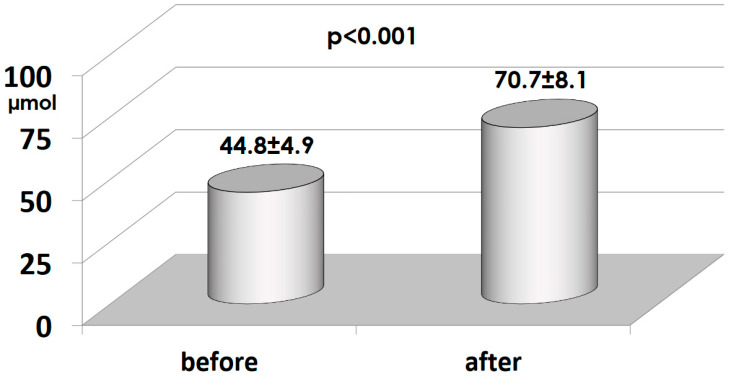
The concentration of nitrite, a stable product of NO metabolism, was measured in the interventricular vein before and after the opening of the LITA-to-LAD graft. The amount of metabolite increased immediately after the opening of LITA [19]. NO, nitric oxide; LITA, left internal thoracic artery; LAD, left anterior descending.

**Figure 3:**
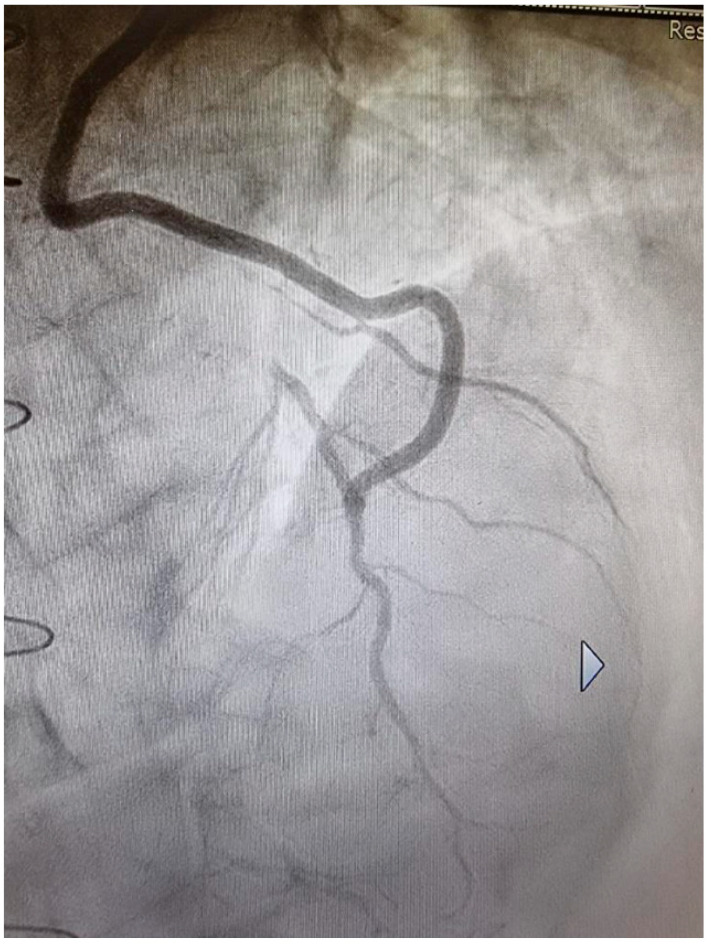
Angiographic control of the LITA, which was anastomosed sequentially to the 1st diagonal and to the LAD, 31 years after surgery. The downstream coronary area is free of atherosclerotic lesions. LITA, left internal thoracic artery; LAD, left anterior descending.

When the SVG is connected to the ITA, either as a Y-graft [12] or as an I-graft [18], many positive effects result. First of all, the LITA is a third-order conduit (aorta, subclavian artery, LITA) and the pulsatile pressure is lower, as in all branched territories. The RITA is a fourth-order conduit (aorta, brachiocephalic artery, subclavian artery, RITA) with an even lower pulsatile pressure than the LITA. The SVG is then exposed to less mechanical stress. However, the most important aspect of this strategy is that the ITAs are drug-eluting grafts, as they produce NO which supplements the coronary arteries [19] (Figure 2) and thus maintains their stability over time (Figure 3). The SVG, when connected to the ITA, may benefit from NO supplementation as the NO enters the coronary circulation via the vein, which can transport the NO to stabilize its ECs. The effect has been shown to become more positive over time. SS in the Y-graft was low shortly after surgery, but increased to arterial levels after 1.5 years [20]. This was due to a change in venous phenotype to arterial. In the SVG, the thickness increased, but to a similar extent as in the LITA [21], and the internal caliber was reduced such that SS increased to arterial levels.

## 4. Conclusions

Even though both surgical strategies showed clinical advantages and the patency rate of SVG increased to a similar or equal value as LITA or RITA after 10 years [13], the Y-graft or I-graft ITA-SVG is in our opinion more advantageous. The SVG is directly supplemented by another conduit that produces high levels of NO, and the coronary circulation is protected by the same NO that protects the SVG. With the no-touch technique, this aspect is not obvious, as it is not certain that the NO produced by the perivascular adipose tissue can reach the lumen of the SVG. With the ITA-SVG connection, NO supplementation by the ITA might be necessary for a few months, not forever as with the no-touch technique, since a true arterialization process could probably be underway and the SVG might act as an AC [20], to restore sufficient NO production to keep the ECs quiet. But this aspect needs to be further explored with multicenter randomized controlled trials. Other factors that may improve the outcome of SVG, such as age, comorbidities and medical treatment, are important, but this article focuses on a metabolic aspect that, together with the elimination of risk factors and appropriate medical therapy, can potentially further improve the results of SVG grafting. Furthermore, the impact of NO on vascular biology is complex and all possible effects need to be better explored. In addition, the impact of possible modifications of the SVG on its long-term outcome has not been studied and needs to be clarified.

To return to our fairy tale, many possibilities have been described for the routes by which the frog became a princess, but in our story, the recently discovered NO seems to play a key role, not only in increasing the rank of SVG, but also in a number of positive effects on our health that we can expect in the near future, when we will be able to fully understand its role in our metabolism.

## Data Availability

Not applicable.
